# Local-to-distant development of the cerebrocerebellar sensorimotor network in the typically developing human brain: a functional and diffusion MRI study

**DOI:** 10.1007/s00429-018-01821-5

**Published:** 2019-02-07

**Authors:** Kaoru Amemiya, Tomoyo Morita, Daisuke N. Saito, Midori Ban, Koji Shimada, Yuko Okamoto, Hirotaka Kosaka, Hidehiko Okazawa, Minoru Asada, Eiichi Naito

**Affiliations:** 1 Center for Information and Neural Networks (CiNet), National Institute of Information and Communications Technology (NICT), 1-4 Yamadaoka, Suita, Osaka 565-0871 Japan; 2 Graduate School of Engineering, Osaka University, 2-1 Yamadaoka, Suita, Osaka 565-0871 Japan; 3 Research Center for Child Mental Development, University of Fukui, 23-3 Matsuoka-shimoaizuki, Eiheiji, Yoshida, Fukui, 910-1193 Japan; 4 Biomedical Imaging Research Center, University of Fukui, 23-3 Matsuoka-shimoaizuki, Eiheiji, Yoshida, Fukui, 910-1193 Japan; 5 Research Center for Child Mental Development, Kanazawa University, 13-1 Takaramachi, Kanazawa, Ishikawa 920-8640 Japan; 6 Graduate School of Engineering Science, Osaka University, 1-3 Machikaneyama, Toyonaka, Osaka 560-8531 Japan; 7 ATR Promotions, 2-2 Hikaridai, Seika, Soraku-gun, Kyoto, 619-0288 Japan; 8 Department of Neuropsychiatry, Faculty of Medical Sciences, University of Fukui, 23-3 Matsuoka-shimoaizuki, Eiheiji, Yoshida, Fukui, 910-1193 Japan; 9 Graduate School of Frontier Biosciences, Osaka University, 1-3 Yamadaoka, Suita, Osaka 565-0871 Japan

**Keywords:** Functional magnetic resonance imaging, Diffusion magnetic resonance imaging, Cerebrocerebellar circuit, Development, Sensorimotor function

## Abstract

Sensorimotor function is a fundamental brain function in humans, and the cerebrocerebellar circuit is essential to this function. In this study, we demonstrate how the cerebrocerebellar circuit develops both functionally and anatomically from childhood to adulthood in the typically developing human brain. We measured brain activity using functional magnetic resonance imaging while a total of 57 right-handed, blindfolded, healthy children (aged 8–11 years), adolescents (aged 12–15 years), and young adults (aged 18–23 years) (*n* = 19 per group) performed alternating extension–flexion movements of their right wrists in precise synchronization with 1-Hz audio tones. We also collected their diffusion MR images to examine the extent of fiber maturity in cerebrocerebellar afferent and efferent tracts by evaluating the anisotropy-sensitive index of hindrance modulated orientational anisotropy (HMOA). During the motor task, although the ipsilateral cerebellum and the contralateral primary sensorimotor cortices were consistently activated across all age groups, the functional connectivity between these two distant regions was stronger in adults than in children and adolescents, whereas connectivity within the local cerebellum was stronger in children and adolescents than in adults. The HMOA values in cerebrocerebellar afferent and efferent tracts were higher in adults than in children (some were also higher than in adolescents). The results indicate that adult-like cerebrocerebellar functional coupling is not completely achieved during childhood and adolescence, even for fundamental sensorimotor brain function, probably due to anatomical immaturity of cerebrocerebellar tracts. This study clearly demonstrated the principle of “local-to-distant” development of functional brain networks in the human cerebrocerebellar sensorimotor network.

**Electronic supplementary material:**

The online version of this article (10.1007/s00429-018-01821-5) contains supplementary material, which is available to authorized users.

## Introduction

Sensorimotor function is a fundamental brain function in humans, and the cerebrocerebellar circuit is essential to this function. Indeed, many resting-state functional connectivity studies have demonstrated that the cerebellum is a member of the sensorimotor network in the adult brain (Buckner et al. 2011; Fox and Raichle 2007; Guell et al. 2018a, b; Habas et al. 2009; Kipping et al. 2013; O’Reilly et al. 2010; Sang et al. 2012).

It is generally believed that the brain regions associated with more fundamental functions may develop earlier than the regions involved in more complex and higher-order functions (Casey et al. 2005; Chugani et al. 1987). For example, it has been demonstrated that, in the human brain, the grey matter maturation of the primary sensorimotor cortex (SM1) occurs relatively earlier in the entire brain, in addition to the occipital visual cortex (Gogtay et al. 2004). Moreover, it has been demonstrated that 6- to 13-year-old children and adolescents already exhibit activation in the cerebrocerebellar sensorimotor network between the contralateral SM1 and the ipsilateral cerebellar hemisphere (lobules V and VI) when they performed finger-tapping tasks with their right hands, with some quantitative difference from adults (De Guio et al. 2012; Turesky et al. 2018). Furthermore, we previously demonstrated that 8- to 11-year-old children recruit the cerebrocerebellar sensorimotor network during kinesthetic (muscle spindle afferent) processing of the right hand (Naito et al. 2017).

On the other hand, it has also been demonstrated that functional connectivity among remote brain regions develops slowly in the human brain (Dosenbach et al. 2010). Resting-state functional connectivity studies have revealed slow maturation of cerebrocerebellar functional networks in general (Fair et al. 2009). According to Fair et al. (2009), the cerebellum and cerebral cortices are functionally connected in the adult brain (19–31 years of age). However, the cerebellum is isolated from the cerebral networks in children (7–9 years of age) and exhibits weak connections with the networks even in adolescents (10–15 years of age). Moreover, such functional connectivity studies have also provided other evidences that the cerebellum is not yet a substantial member of the sensorimotor network in neonates (Fransson et al. 2007) or young (2- to 5-year-old) children (Manning et al. 2013), becoming a member in 5- to 8-year-old children (de Bie et al. 2012) as shown in adults (see above references). Thus, it remains controversial whether the cerebellum is a substantial member of the sensorimotor network in children and adolescents. In addition, these evidence are derived from examining functional connectivity while participants are resting without performing any motor tasks; thus, it is unclear whether children and adolescents (approximately 8–15 years of age) exhibit adult-like functional connectivity in the cerebrocerebellar sensorimotor network when they actually perform a motor task. If we consider the fact that functional brain networks develop slowly and from a local to distributed organization (Dosenbach et al. 2010; Fair et al. 2007, 2009), we may expect that, during a motor task, functional connectivity in the cerebrocerebellar distant network is still under development even for fundamental sensorimotor function in children, and that there could be a developmental shift from local to long-range connectivity in the cerebrocerebellar sensorimotor network from childhood to adulthood.

In the present study, we first conducted functional magnetic resonance imaging (fMRI) in a total of 57 right-handed healthy children (aged 8–11 years), adolescents (12–15 years), and young adults (18–23 years; 19 per group). We scanned their brain activity while the blindfolded participants performed alternating extension–flexion movements of their right wrists in precise synchronization with 1-Hz audio tones. In addition to conventional contrast analyses to identify group-specific and common-across-groups activations, we conducted a seed-based functional connectivity analysis to explore possible group-specific neuronal communication patterns in the sensorimotor network.

We then conducted diffusion MRI in which we collected diffusion-weighted MR images from these participants to examine the extent of fiber (anatomical) maturity of their cerebrocerebellar tracts. Despite that abnormalities in the development of the cerebellar white matter have been relatively well-documented (Catani et al. 2008; Fatemi et al. 2012), the development of cerebellar afferent and efferent tracts in the typically developing human brain (from childhood to adulthood) has not been fully elucidated. This is important because such an effort can provide valuable information about normal cerebrocerebellar anatomical development which may underpin its functional development.

In primates, the cerebellar hemispheres receive afferent inputs from the contralateral cerebral cortices and project efferent outputs back to these cortices by forming parallel cortico-ponto-cerebello-dentato-thalamo-cortical closed loops (Clower et al. 2005; Dum and Strick 2003; Kelly and Strick 2003; Middleton and Strick 2000; Strick et al. 2009). These pathways are referred to as the cerebrocerebellar tracts [the cortico-ponto-cerebellar (CPC) afferent tract and the superior cerebellar efferent (SC; cerebello-dentato-thalamo-cortical) tract]. Thus, we focused on these cerebrocerebellar afferent and efferent tracts and evaluated the extent of fiber maturity of these tracts in each participant, and examined possible group differences. We also discussed relationship between the development of functional connectivity in the cerebrocerebellar sensorimotor network and the anatomical maturation of the cerebrocerebellar tracts.

## Materials and methods

### Participants

A total of 57 healthy volunteers participated in the study. The participants were composed of three (child, adolescent, and adult) groups. The child (CH) group consisted of 19 children (mean age 9.5 ± 0.9 years, range 8 years 7 months to 11 years 3 months). The adolescent (ADO) group was composed of 19 adolescents (mean age 13.4 ± 0.7 years, range 12 years 8 months to 15 years 0 months). The adult (AD) group consisted of 19 young adults (mean age 20.8 ± 1.4 years, range 18 years 10 months to 23 years 7 months). The children and adolescents were recruited from local elementary and junior-high schools. We confirmed handedness using the Edinburgh Handedness Inventory (Oldfield 1971), and ensured that no participants had a history of neurological or psychiatric disorder based on self and legal guardian reports. The protocol used in the study was approved by the ethics committees of the University of Fukui and the National Institute of Information and Communications Technology. We explained the details of the study to the participants before initiating the experiment. After this explanation, all participants provided written informed consent. In cases of children and adolescents, written informed consent was also obtained from their legal guardians. The experiment was carried out following the principles and guidelines of the Declaration of Helsinki (1975).

### Motor task

Before the fMRI experiment, each participant performed a motor task outside the scanner to familiarize them with the task before they entered the MR room. The participants subsequently lay in the MRI scanner. At this time, their heads were immobilized using sponge cushions and their ears were plugged. Both the left and right arms of the participants were naturally semipronated and extended in front of them. Both arms were supported by cushions, allowing the participants to relax their upper arms during the motor task. We asked the participants to relax their entire body without producing unnecessary movements and to think only of the assigned tasks.

**Fig. 1:**
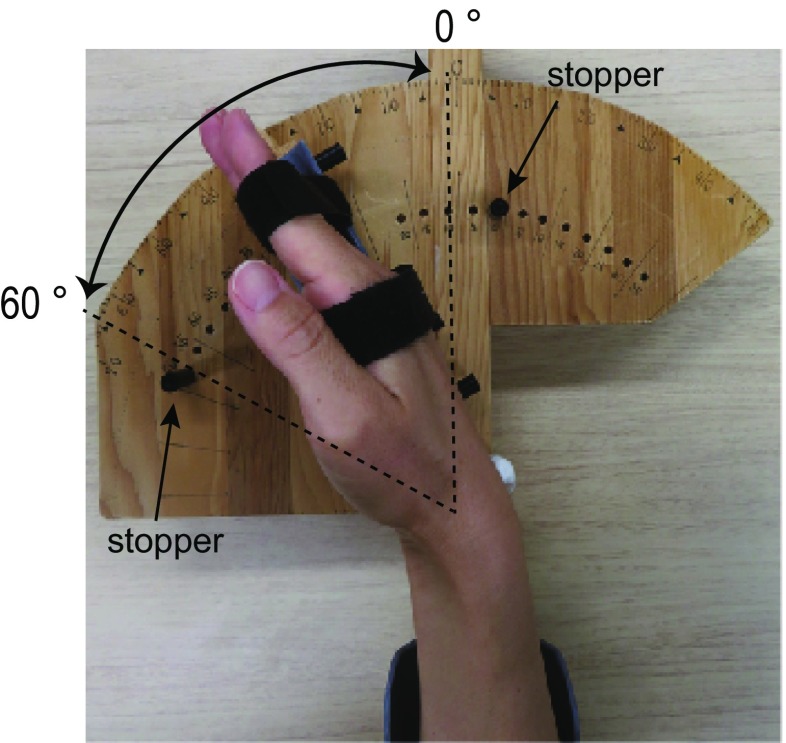
Experimental setup for a motor task. The blindfolded participants were asked to perform alternating extension–flexion movements of their right wrists in precise synchronization with 1-Hz audio tones generated by a computer. Their right hands were affixed to a wooden apparatus. We fixed the right hand on a mobile indicator with two hook and loop fasteners in order for the radiocarpal joint of the wrist to be located immediately above the origin of the protractor. We defined the angle of the wrist as 0° when the wrist was straightened as the start position. To control the range of wrist motion across epochs and participants, we fixated two stoppers [the straight (0°) position and 60° of flexion] on the protractor device. The participants had to touch either stopper (at 0° or 60°) by the hand-fixed mobile indicator in precise synchronization with 1-Hz audio tones, while they kept controlling the alternating wrist extension–flexion movements

Their right hands were affixed to a wooden apparatus (Fig. 1). A mobile indicator was mounted on the surface of this apparatus, and angular degrees were scaled using an ordinal protractor on its surface. We fixed the right hand on this mobile indicator with two hook and loop fasteners, while the index, middle, ring, and little fingers were extended. Special care was taken to ensure that the two fasteners were wrapped around the hands consistently across the participants, to match the areas that received tactile inputs from the fasteners among the participants. One fastener wrapped the proximal interphalangeal joints, and the other wrapped the metacarpal bones (Fig. 1). The radiocarpal joint of the wrist was located immediately above the origin of the protractor. We defined the wrist angle as 0° when the wrist was straightened as the start position (Fig. 1).

The blindfolded participants were asked to perform alternating extension–flexion movements of their right wrists in precise synchronization with 1-Hz audio tones generated by a computer. We fixated two stoppers on the protractor device to control the range of wrist motion across epochs and participants (Fig. 1). One was fixated at the start position to prevent the wrist from extending beyond the straight (0°) position. The other was prepared to prevent the wrist from flexing beyond 60° of flexion. The participants had to touch either stopper (0° or 60°) by the hand-fixed mobile indicator in precise synchronization with 1-Hz audio tones, while making controlled alternating wrist extension–flexion movements. Hence, they had to continuously control wrist extension and flexion movements alternatingly by predicting and adjusting movement time within the range of motion (from 0° to 60°), and thus the task required predictive sensory-motor control and online feedback control of the wrist.

Each participant completed one experimental run, which lasted for 205 s. One run was composed of six movement epochs (Move), each of which lasted for 15 s. The movement epochs were separated by 15 s resting (baseline) periods. During the resting periods (Rest), 1-Hz audio tones were generated at different pitches. Thus, during the resting periods, the participants received 1-Hz auditory stimuli but did not move their right wrists. Each run also included a 25 s period before the start of the first epoch and another 15 s period after the end of the final epoch.

We asked the participants to close their eyes immediately before we started an fMRI run. During the fMRI run, we provided the participants with auditory instructions (e.g. 3, 2, 1, start) through an MR-compatible headphone to provide them the start timing of a movement epoch. We also provided the instruction “stop” to notify the participants of the cessation time for each movement epoch. These instructions were also generated by a computer. We confirmed that all of the participants could perform the 1 Hz movements by visual inspection during scanning.

### fMRI data acquisition

MR images were acquired using a 3.0 T Discovery MR 750 scanner (General Electric Medical Systems, Milwaukee, Wisconsin, USA). The methods of fMRI data acquisition were identical to those in our previous studies (Morita et al. 2018; Naito et al. 2017). Functional images were acquired using T2*-weighted gradient echo-planar imaging (EPI) sequences obtained using the MRI machine and a 32-channel array coil. We collected 82 volumes per run [slice number = 40; slice thickness = 3.5 mm; inter-slice thickness = 0.5 mm; repetition time (TR) = 2500 ms; echo time (TE) = 30 ms; flip angle = 83°; field of view (FOV) = 192 × 192 mm; voxel size (*x, y, z*) = 3 × 3 × 4 mm].

### Imaging data analysis

#### Preprocessing

To eliminate the effects of unsteady magnetization in the task, we discarded the first four EPI images in the fMRI run. Imaging data were analyzed using SPM 8 (The Wellcome Trust Centre for Neuroimaging, London, UK) implemented in Matlab (Mathworks, Sherborn, MA).

Initially, the EPI images were realigned to the first image and then to the mean image. We first calculated the mean displacement of each image from the first image for each run with each participant. All 57 participants had less than 3 mm of cut-off maximum motion in every plane (*x, y, z*) during the run. When we computed the average displacement across participants in each group (Morita et al. 2018), we found that in the CH group the average displacements were 0.08 mm (range 0.02–0.25 mm), 0.13 mm (0.14–0.44 mm), and 0.31 mm (0.03–1.90 mm) in the *x*-, *y*-, and *z*-axes, respectively. In the ADO group, these values were 0.07 mm (0.02–0.15 mm), 0.13 mm (0.03–0.48 mm), and 0.17 mm (0.04–0.34 mm). In the AD group, these values were 0.09 mm (0.02–0.20 mm), 0.10 mm (0.02–0.43 mm), and 0.10 mm (0.04–0.28 mm). A one-way ANOVA revealed no significant differences across groups in any axes.

The realigned images were normalized to the Montreal Neurological Institute (MNI) space (Evans et al. 1994). By comparing the functional activation foci in children and adults within a common stereotaxic space, Kang et al. (2003) provided an empirical validation of normalization for analysis of fMRI data obtained from school-aged children (Kang et al. 2003). Finally, the spatially normalized functional images were filtered using a Gaussian kernel with a full-width-at-half-maximum (FWHM) of 4 mm along the *x*-, *y*-, and *z*-axes.

### Analysis of movement-related activation in each group

After preprocessing, we first explored movement-related activation using a general linear model (GLM; Friston et al. 1995; Worsley and Friston 1995) in each participant. The design matrix contained a boxcar function for the movement epoch, which was convolved with a canonical hemodynamic response function. To correct for residual motion-related variance after realignment, the six realignment parameters were also included in the design matrix as regressors of no interest.

We first generated a contrast image to examine the brain regions that exhibited movement-related activation (Move > Rest) in each participant (single-subject analyses). In this contrast, the effect of 1-Hz audio tones should be eliminated because the participants consistently heard the 1-Hz audio tones both in the movement epochs and in the resting periods. The contrast images from all participants were entered into a second-level random effects group analysis (Holmes and Friston 1998). One-sample *t* tests were conducted in each group separately. In the second-level analyses, we first generated a voxel-cluster image using an uncorrected voxel-wise threshold of *p* < 0.001 in each group. For statistical inference, we used an extent threshold of *p* < 0.05 at the cluster level after correction for multiple comparisons with the family-wise error (FWE) rate in the whole brain.

To identify the anatomical regions of activation peaks, we referred to the cytoarchitectonic probability maps in the MNI standard brain of the SPM Anatomy Toolbox v2.2b (Eickhoff et al. 2005). With regard to the definition of cerebellar regions, we referred to the study by Schmahmann et al. (2000) when anatomical definition was not available in the toolbox.

### Consistent brain activation across groups

To explore the brain regions that consistently exhibited movement-related activation across all age groups, we performed a conjunction analysis (Price and Friston 1997). In this analysis, we also generated a voxel-cluster image using an uncorrected voxel-wise threshold of *p* < 0.001, and used the FWE-corrected cluster-wise threshold of *p* < 0.05 in the entire brain space.

### Comparisons between groups

To examine developmental changes in brain activation, we examined all possible comparisons across three groups. For example, when we examined the greater activation in the AD group relative to the CH group, we compared movement-related activation in the AD group with that in the CH group by examining (Move > Rest)_AD_ − (Move > Rest)_CH_. In this comparison, we used an image of (Move > Rest)_AD_ (uncorrected height threshold of *p* < 0.05) as an inclusive mask. Using this masking procedure, we ensured that any movement-related activation that we observed to be greater in the AD group was true activation in the AD group, rather than pseudo-activation caused merely by movement-related deactivation in the CH group. We used the same procedure for other comparisons. The validity of the masking procedure is discussed in our previous papers (Morita et al. 2018; Naito et al. 2017). In these group comparisons, we used the FWE-corrected extent threshold of *p* < 0.05 in the entire brain for a voxel-cluster image generated with an uncorrected cluster-defining height threshold of *p* < 0.001 in each comparison.

### Functional connectivity analysis

We conducted a functional connectivity analysis to examine possible group differences in functional connectivity in the sensorimotor network. Specifically, we expected a developmental shift from local to long-range connectivity in the cerebrocerebellar sensorimotor network from childhood to adulthood (see “Introduction”).

**Fig. 2:**
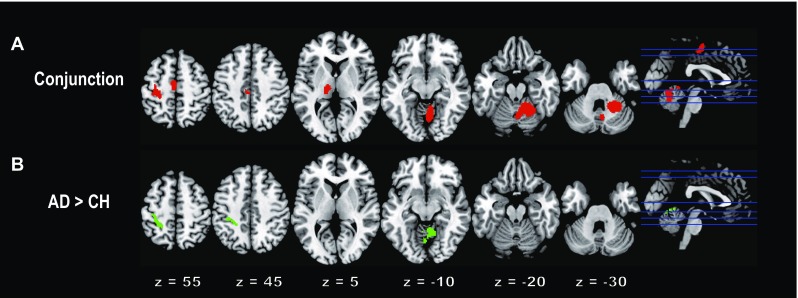
**a** Results of the conjunction analysis. Brain regions that were consistently activated during the motor task across all (CH, ADO, and AD) groups are indicated in red. **b** Results of the between-group comparison. Brain regions in which the AD group exhibited significantly greater movement-related activation than did the CH group are indicated in green. In each panel, brain activations are rendered onto the horizontal slices of the Montreal Neurological Institute standard brain (slices: *z* = 55, 45, 5, − 10, − 20 − 30 from left to right). Each slice level is indicated by a blue horizontal line on the brain depicted at the rightmost side in each panel. *AD* adult, *ADO* adolescent, *CH* child

The conjunction analysis revealed consistent movement-related brain activation across all age groups in the contralateral SM1, supplementary motor area (SMA), cingulate motor area (CMA), and thalamus and in the ipsilateral cerebellar vermis, paravermis, and hemisphere (Table 1; Fig. 2a). Highly similar patterns of brain activation were identified in our previous study in which an independent group of blindfolded healthy adults performed similar right-wrist movements (Amemiya and Naito 2016). Thus, such active brain regions can be considered important regions during right-wrist movements. In this previous study, we reported 15 peak voxels in such active brain regions. In the present study, we confirmed that 7 out of these 15 voxels were also active in the conjunction analysis. These seven voxels were located in the right lobule VI (two voxels), right paravermis (lobule V), left area 4a, left SMA, left CMA, and left thalamus. We used each of these peak voxels in the following connectivity analysis as a seed voxel. By selecting seed voxels based on the results obtained from the previous independent study (Amemiya and Naito 2016), we avoided any problematic circular evaluation raised by Kriegeskorte et al. (2009). In the present study, the primary somatosensory cortex (SI) was not included as a seed region. This is because, although we found an activation peak (− 36, − 40, 68) in SI in our previous study (Amemiya and Naito 2016), the present common SM1 activation was predominantly located in the primary motor cortex (M1), and thus this peak was not identified in the present common SM1 activation.

**Table 1:** Results of conjunction analysis

Clusters	Size	*t* value	*x*	*y*	*z*	Area
Right cerebellar cluster	1630	10.41	14	− 46	− 22	Lobule V (Hem)
		9.38	24	− 46	− 28	Lobule VI (Hem)
		9.21	4	− 62	− 26	Lobule VI (Verm)
		6.61	6	− 68	− 38	Lobule VIIIa (Verm)
		4.66	22	− 58	− 50	Lobule VIIIb (Hem)
Left M1 cluster	754	8.11	− 34	− 26	60	Area 4a
		5.43	− 32	− 24	72	Precentral gyrus
		5.07	− 20	− 26	78	Postcentral gyrus
Left thalamic cluster	200	6.45	− 16	− 22	4	Thalamus
Left medial frontal cluster	227	5.61	− 6	− 20	50	CMA

Uncorrected height threshold, *p* < 0.001; extent threshold, *p* < 0.05, FWE-corrected in the entire brain. Size = number of active voxels. For anatomical identification of peaks, we only considered cytoarchitectonic areas available in the Anatomy toolbox that had > 30% probabilities. The cytoarchitectonic area with the highest probability was reported for each peak. When no cytoarchitectonic area with a > 30% probability was available to determine a peak, we provided the anatomical location of the peak. In each cluster, we reported peaks that were more than 8 mm apart in the descending T-value order. To facilitate visualization, we avoided reporting a peak for each cluster when it was identified in the cytoarchitectonic area or anatomical structure already reported for a peak with a higher *t* value

*Hem* hemisphere, *Verm* vermis, *CMA* cingulate motor area

The time-course data were extracted from the 8 mm radius sphere around each peak (seed voxel) in each participant. This radius was selected based on the final smoothness of the present functional imaging data. In this analysis, we constructed a second GLM (independent of the first GLM) for each seed region in each participant. The time-course data obtained from a seed voxel were included as a linear regressor in the design matrix for the GLM. This GLM also contained a movement-related regressor for the movement epoch, and the six realignment parameters, as regressors of no interest. In general, during the performance of a particular task, the blood oxygen-level dependent (BOLD) signal includes both task-related and neuronal fluctuation components (Saito et al. 2010). Thus, the regressor (the time-course data obtained from a seed voxel) most likely contained both task-related and neuronal fluctuation components. In the present connectivity analysis, we expected to identify brain regions in which activity co-varied with activity changes (movement-related and neuronal fluctuation components) in a seed region, which cannot be detected merely by the movement-related regressor (simple boxcar function). Hence, the brain regions detected in this analysis were likely to share these activity components with the seed region.

**Fig. 3:**
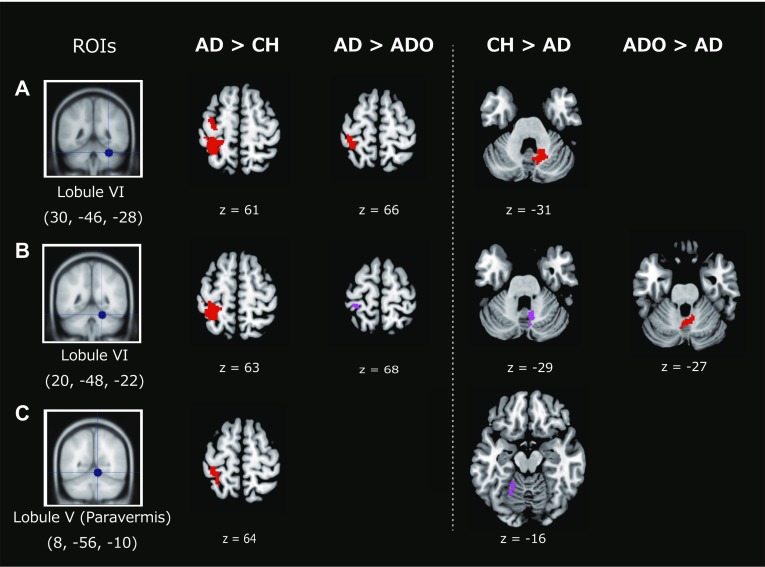
Results from functional connectivity analyses. **a**–**c** Represents the brain regions in which the functional connectivity with each seed region [leftmost column: regions of interest (ROIs)] was greater in one group than in another group. For example, when the connectivity was greater in the AD group than in the CH group, we described this as AD > CH. We demonstrated between-group differences in connectivity when we used a seed region of **a** right lobule VI [Montreal Neurological Institute (MNI) coordinates (30, − 46, − 28)], **b** right lobule VI (20, − 48, − 22), and **c** right paravermis lobule V (8, − 56, − 10). Each seed region (blue dot) was rendered onto a coronal slice of the MNI standard brain. The brain regions that exhibited significant between-group differences in connectivity were superimposed on horizontal slices of the MNI standard anatomical image (family-wise error-corrected spatial-extent threshold of *p* ≤ 0.05 in the entire brain; red sections). The *z*-coordinate of the horizontal slice is indicated immediately below each panel. To demonstrate the coherency of our results, we also present the brain regions in which we found clusters of active voxels (> 45 voxels) that survived at the cluster-defining uncorrected height threshold of *p* < 0.001 (violet sections). *AD* adult, *ADO* adolescent, *CH* child

The above analysis generated an individual image, which identified the brain regions where activity co-varied with the activity in each seed region. The images obtained from all participants were entered into the second-level random effects group analysis. This was carried out in each age group separately. We then conducted the second-level analyses by performing two-sample *t* tests between any two given groups. In this comparison, we used the average image of (Move > Rest) across all age groups (uncorrected height threshold of *p* < 0.05) as an inclusive mask. Using this masking procedure, we ensured that any brain regions exhibiting functional connectivity that we observed to be stronger in an age group was restricted to brain regions that exhibited movement-related activation observed across all groups on average. For statistical evaluation, we used the FWE-corrected extent threshold of *p* < 0.05 in the entire brain for a voxel-cluster image generated at the cluster-defining uncorrected height threshold of *p* < 0.001. We found significant group differences only when we used the cerebellar seeds, but not from area 4a, SMA, CMA, or thalamic seeds. Hence, we only report the results obtained from the cerebellar seed regions (red sections in Fig. 3). To verify the consistency of the present results, we also examined brain regions in which we found clusters of active voxels (> 45 voxels) that survived at the cluster-defining uncorrected height threshold of *p* < 0.001 (violet sections in Fig. 3). This was a purely descriptive approach; however, this may help us to demonstrate the coherency of the present results (Fig. 3).

**Fig. 4:**
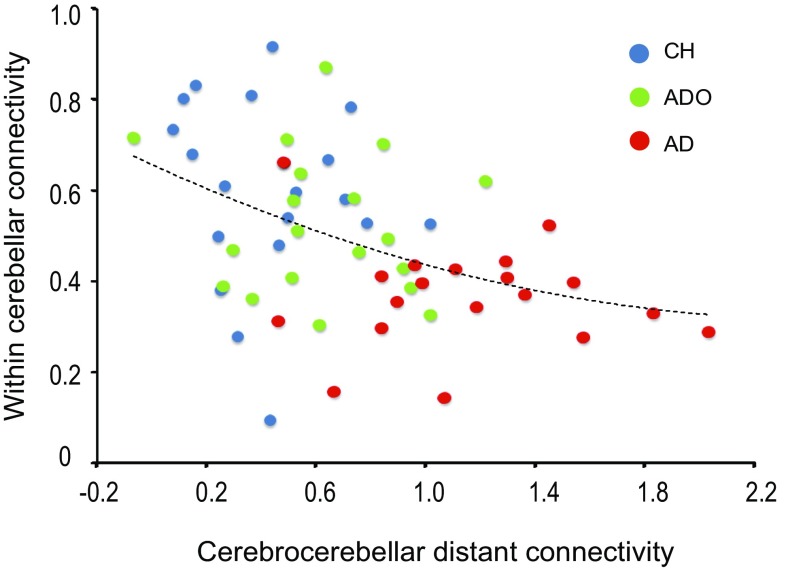
“Local-to-distant” development of functional connectivity in the human cerebrocerebellar sensorimotor network during a motor task. We plotted individuals’ effect-sizes of within-cerebellar connectivity (vertical axis) against their cerebrocerebellar distant connectivity (horizontal axis). The blue, green, and red dots represent data obtained from each participant in the CH, ADO, and AD groups, respectively. The dotted curve indicates a regression fitting when we applied a quadric function to the data obtained from all groups. These data indicate that the human cerebrocerebellar sensorimotor network develops by reducing within-cerebellar local connectivity and increasing long-range cerebrocerebellar connectivity. *AD* adult, *ADO* adolescent, *CH* child

In the functional connectivity analysis, we found that adults exhibited stronger connectivity in the long-range cerebrocerebellar network, whereas children and adolescents exhibited more local connectivity within the cerebellum (Fig. 3). To visualize this “local-to-distant” development of the cerebrocerebellar sensorimotor network, we examined developmental changes in the effect-size of functional connectivity with the seed region of the right cerebellar hemisphere [lobule VI; coordinates (20, − 48, − 22)]. We selected this seed region because only from this seed region could we identify clusters of active-voxels (> 45 voxels at the uncorrected height threshold of *p* < 0.001) that exhibited stronger functional connectivity consistently in the between-group comparisons of AD vs. CH, AD vs. ADO, CH vs. AD, and ADO vs. AD. In each participant of all groups, we first extracted the effect-size of long-range cerebrocerebellar connectivity from the 8 mm radius sphere around a peak voxel [postcentral gyrus: (− 30, − 36, 70)] of the active-voxel cluster, which exhibited stronger functional connectivity in the AD group consistently when compared to the CH and ADO groups (left two panels in Fig. 3b). We also extracted the effect-size of the within-cerebellar connectivity from the 8 mm radius sphere around a peak voxel [interpositus nuclei: (6, − 58, − 28)] of the active-voxel cluster, which exhibited stronger functional connectivity in the CH and ADO groups than in the AD group (right two panels in Fig. 3b). We then plotted individual within-cerebellar connectivity against cerebrocerebellar distant connectivity (Fig. 4). This visualization approach could improve our understanding of the “local-to-distant” developmental dynamics of the human cerebrocerebellar sensorimotor network.

### Diffusion MRI data acquisition

MR images were acquired using the same 3.0 T scanner. During scanning, the participants’ heads were immobilized using sponge cushions, and their ears were plugged. We asked the participants to relax their entire body and avoid moving. A silent cartoon video was projected to the participants during the 4 min 29 s scanning time to keep them still in the scanner (Greene et al. 2016). Diffusion-weighted MR images were acquired using a spin-echo EPI sequence taken in the axial plane with 30 directions, with a b value of 1000 s/mm^2^. The image consisted of 64 slices acquired in ascending order, with a thickness of 2 mm. The time interval between two successive acquisitions from the same slice (TR) was 8400 ms. We used a TE of 90.5 ms and a flip angle of 90°. The FOV was 256 × 256 mm, and the matrix size was 256 × 256. Eventually, the voxel dimensions were 1 × 1 × 2 mm in the *x*-, *y*-, and *z*-axes. Additionally, a single image with no diffusion weighting (*b* value = 0 s/mm^2^; b0 image) was acquired.

### Data analyses

#### General procedures

In the series of analyses, we first corrected for head motions and geometrical distortions in the diffusion-weighted MR image (preprocessing). We then estimated the distribution of fiber orientations in each voxel of the entire brain using a spherical deconvolution (SD) approach (Tournier et al. 2004). We reconstructed the streamlines (estimated trajectories of white matter fascicles) in the entire brain (tractography). We defined multiple regions-of-interest (ROIs) to dissect two targeted tracts (the CPC afferent tract and the SC efferent tract) from all of the streamlines. For each tract, the tract connecting the right cerebellum and the left cerebrum and that connecting the left cerebellum and the right cerebrum were treated separately. To evaluate tract tissue properties, we calculated hindrance modulated orientational anisotropy (HMOA) as an anisotropy index, which has been demonstrated to more sensitively and precisely detect subtle changes in water diffusivity associated with complex fibers (Dell’Acqua et al. 2013). We calculated the HMOA values for the CPC tract and the SC tract of each hemisphere (right-cerebellum–left-cerebrum and left-cerebellum–right-cerebrum) in each participant and examined the differences among the three (CH, ADO, and AD) groups.

### Preprocessing

We used freely accessible software (Explore DTI; http://www.exploredti.com: Leemans et al. 2009) to register raw diffusion-weighted MR images for each participant, and corrected for head motions and geometrical distortions in each slice of the image.

### SD approach and tractography

After preprocessing, we used an SD approach to estimate the orientation distribution of fiber populations within individual voxels. The limitation of the conventional tensor model-based approach [diffusion tensor imaging (DTI)] is the inability to identify multiple fiber orientations within a voxel and to solve crossing fibers during tracking. In contrast, the SD approach allows for the estimation of multiple fiber orientations within a voxel based on spherical harmonics, and thus provides higher sensitivity for identifying white matter tracts even in cases of fiber crossing (Dell’Acqua et al. 2013; Tournier et al. 2004). Since the complex distribution of fiber orientation is highly probable in the developing brain, the SD approach is considered the more appropriate approach for evaluating the maturity of white matter fibers in such brains (Dell’Acqua et al. 2013). Therefore, we opted to use the SD approach in this study to improve the sensitivity of tract identification.

The SD was performed using a modified (damped) version of the Richardson–Lucy algorithm (Dell’acqua et al. 2010) implemented in StarTrack software (http://www.natbrainlab.com). We selected a fixed fiber response that corresponded to a shape factor of *α* = 2 × 10^− 3^ mm^2^/s (Dell’Acqua et al. 2013). To reduce the partial volume effect of isotropic compartments (gray matter or cerebrospinal fluid), we applied an absolute threshold of 0.1 and a relative threshold of 10%. Based on these voxel data, we performed SD-based fiber tracking using a modified Euler integration algorithm (Dell’Acqua et al. 2013) to generate streamlines in the entire brain. Each streamline was halted when it reached a voxel with no specific fiber orientation or when a fiber orientation identified in a voxel exceeded 45° from the direction of the fiber streamline reconstructed via the voxels that were processed one step before. These analyses were performed for each participant.

### ROI setting

We then dissected our target fiber tracts from the streamlines in the entire brain (whole-brain tractography). In the present study, we focused on two target tracts (the CPC afferent tract and the SC efferent tract). For each tract, the right-cerebellum–left-cerebrum tract and the left-cerebellum–right-cerebrum tract were reconstructed separately. To dissect these tracts, we imported the whole-brain tractography into TrackVis software (http://www.trackvis.org: Wang et al. 2007). We defined ROIs for each tract. The ROIs were manually drawn based on a previous study (Catani et al. 2008). With respect to the CPC tract, we set the ROIs around the middle cerebellar peduncle and the contralateral cerebral peduncle. By dissecting the streamlines that passed through these ROIs, we were able to reconstruct the major afferent pathway from the contralateral cerebral cortex to the cerebellar hemisphere via the pontine nuclei, i.e. the CPC tract. With respect to the SC tract, we set the ROIs around the cerebellar nuclei and the central part of the SC peduncle. We assumed that this tract included both the dentato-(rubro)-thalamic tract and the cerebellar-rubro-olivo-cerebellar network (Catani et al. 2008; Catani and Thiebaut de Schotten 2012).

### Calculation of the HMOA value

Finally, we calculated the HMOA values from each reconstructed tract in each participant to estimate the tissue properties along the tract. The HMOA is an index of white matter diffusivity, indicating the normalized anisotropy of diffusion signal estimated by the SD model along the orientation of streamlines that belong to the tract of interest (range 0–1). A value of 1 is the highest possible value indicating the diffusion of water-molecules that is restricted to a single axis, whereas a value of 0 indicates the absence of a fiber.

The advantage of HMOA is that we could separately estimate the diffusivity along different fiber tracts that cross within a voxel, unlike tensor-based metrics (e.g. FA), and this ensures a more sensitive analysis. In this manner, HMOA enables a more precise approximation of the distribution of complex fiber orientations. Indeed, simulation analyses have demonstrated that HMOA values can sensitively reflect smaller changes in fiber diffusivity than can the fractional anisotropy (FA) value (Dell’Acqua et al. 2013). Hence, these values may describe the microstructural organization of white matter fibers (degree of myelination, axon density, axon diameter, or fiber orientation dispersion etc.) more precisely and sensitively in the developing brain.

We calculated the HMOA value for each tract in each participant. For the statistical evaluation of group differences in HMOA values, we performed a one-way ANOVA. For the post hoc test, we used multiple comparisons of Tukey’s test. The significance threshold was set to *p* < 0.05 (FWE corrected for multiple comparisons).

## Results

### fMRI

All participants could perform the movements of the right wrist at 1 Hz. When we examined movement-related activity in each age group, we found a similar pattern of brain activation across all groups. Indeed, the conjunction analysis revealed consistent brain activation across all age groups in the hand/arm section of the contralateral (left) SM1 (mainly in M1: cytoarchitectonic area 4a), SMA, CMA, and thalamus, and in the ipsilateral (right) cerebellar vermis, paravermis, and hemisphere (lobules V, VI, VIIIa, and VIIIb; Table 1; Fig. 2a). Among these cerebellar regions, lobules V and VI likely correspond to the right hand/arm sections of the cerebellum that form the cerebrocerebellar motor network with the contralateral M1 (Naito et al. 2017).

Despite this consistency, we found greater activity in the contralateral (left) SM1 (areas 4p, 3a, 1) extending into the superior parietal lobule (SPL; area 5L) and in the ipsilateral (right) cerebellar vermis, paravermis, and hemisphere (lobules V and VI) in the AD group than in the CH group (Table 2; Fig. 2b). Importantly, the greater activity was not evident in the anterior portion of M1 (area 4a), but was caudally located mainly in the postcentral gyrus and in the cortices lining the postcentral sulcus, which can be considered higher-order somatosensory and sensorimotor-association areas (Fig. 2b). We could not find any significant group difference in other possible comparisons.

**Table 2:** Greater movement-related brain activity in AD group compared to CH group

Clusters	Size	*t* value	*x*	*y*	*z*	Area
Left SM1 cluster	458	5.88	− 32	− 36	68	Area 1
		5.49	− 26	− 42	56	Area 5L (SPL)
		4.16	− 30	− 36	52	Area 3a
		3.91	− 38	− 24	52	Area 4p
		3.51	− 18	− 32	76	Postcentral gyrus
Bilateral cerebellar cluster	239	5.20	10	− 52	− 12	Right lobule V (Hem)
		3.88	− 2	− 66	− 8	Left lobule VI (Verm)
		3.60	− 6	− 54	− 8	Left lobule V (Hem)

See footnote in Table 1

*AD* adults, *CH* children, *SPL* superior parietal lobule, *Hem* hemisphere, *Verm* vermis

### Functional connectivity

#### Stronger functional connectivity in adults

When we examined the brain regions in which activity co-varied with that in the ipsilateral (right) cerebellar hemisphere [lobule VI; coordinates (30, − 46, − 28)], we found the contralateral (left) SM1 (areas 4a, 3a, 3b, and 1), higher-order somatosensory area (area 2), and SPL (area 5L) exhibited stronger functional connectivity in the AD group than in the CH group (Table 3 and the leftmost panel in Fig. 3a). We also found highly similar results in the AD group relative to the ADO group (Table 3 and the left-middle panel in Fig. 3a).

**Table 3:** Brain regions in which AD group showed stronger functional connectivity with each seed region

Seed region	Contrast	Clusters	Size	*t* value	*x*	*y*	*z*	Area
Lobule VI (Hem) (30, − 46, 28)	AD > CH	Left SM1 cluster	1085	7.13	− 30	− 36	70	Postcentral gyrus
				6.18	− 40	− 16	50	Area 4a
				5.79	− 36	− 42	64	Area 1
				5.31	− 40	− 26	52	Area 3b
				4.86	− 32	− 12	64	Precentral gyrus
				4.77	− 30	− 36	52	Area 3a
				3.95	− 26	− 44	54	Area 5L (SPL)
	AD > ADO	Left SM1 cluster	441	6.14	− 30	− 36	70	Postcentral gyrus
				5.41	− 40	− 16	50	Area 4a
				5.27	− 40	− 26	52	Area 3b
				4.63	− 50	− 24	54	Area 1
				4.52	− 38	− 40	62	Area 2
				3.97	− 24	− 36	52	Area 5L (SPL)
				3.84	− 26	− 46	70	Precentral gyrus
Lobule VI (Hem) (20, − 48, − 22)	AD > CH	Left SM1 cluster	812	7.29	− 30	− 36	70	Postcentral gyrus
				5.39	− 34	− 42	64	Area 1
				4.76	− 28	− 50	64	Area 5L (SPL)
				4.71	− 16	− 32	76	Area 4a
				4.36	− 22	− 22	68	Precentral gyrus
				4.36	− 30	− 36	52	Area 3a
		Left SI cluster	165	6.14	− 42	− 14	52	Postcentral gyrus
				4.29	− 48	− 28	54	Area 1
Lobule V (Hem) (Paravermis) (8, − 56, − 10)	AD > CH	Left SI cluster	515	5.08	− 36	− 36	66	Area 1
				3.58	− 28	− 52	64	Area 7A (SPL)

See footnote in Tables 1 and 2

A highly similar pattern of stronger functional coupling in the AD group relative to the CH group was also observed when we used another cerebellar seed region of lobule VI [coordinates (20, − 48, − 22); Table 3 and the leftmost panel in Fig. 3b). As for this seed region, we also found two clusters of active voxels in the contralateral SI [48 voxels in area 1, peak coordinates = (− 30, − 36, 70)] and M1 [46 voxels in area 4a (− 40, − 14, 50)], which exhibited greater functional coupling in the AD group than in the ADO group (violet sections in left-middle panel in Fig. 3b).

In favor of the above results, when we computed the connectivity from the ipsilateral paravermal region [lobule V; coordinates (8, − 56, − 10)], we found stronger functional coupling of the contralateral SI (area 1) extending into the SPL (area 7A) in the AD group than in the CH group (Table 3 and the leftmost panel in Fig. 3c).

Viewed collectively, the series of results indicated that activity in the right cerebellum was more strongly coupled with that in the left cerebral cortices (SM1, higher-order somatosensory and sensorimotor-association areas) in the AD group than in the younger (CH and ADO) groups, indicating stronger functional connectivity in the long-range cerebrocerebellar sensorimotor network during the motor task in adults.

#### Stronger functional connectivity in children and adolescents

We observed different patterns of functional coupling in the CH and ADO groups when compared to those in the AD group. When we examined the brain regions in which activity co-varied with that in the ipsilateral (right) cerebellar hemisphere [lobule VI; coordinates (30, − 46, − 28)], we found stronger functional coupling within the cerebellum (peaks in the right dentate and interpositus and fastigial nuclei) in the CH group than in the AD group (Table 4 and the right-middle panel in Fig. 3a).

**Table 4:** Brain regions in which younger groups showed stronger functional connectivity with each seed region

Seed region	Contrast	Clusters	Size	*t* value	*x*	*y*	*z*	Area
Lobule VI (Hem) (30, − 46, − 28)	CH > AD	Right cerebellar cluster	173	4.64	20	− 58	− 32	Dentate nucleus
				4.43	12	− 64	− 30	Interpositus nuclei
				3.93	6	− 58	− 26	Fastigial nucleus
Lobule VI (Hem) (20, − 48, − 22)	ADO > AD	Right cerebellar cluster	89	5.09	2	− 56	− 26	Fastigial nucleus

See footnote in Tables 1 and 2. For the definition of cerebellar regions, we referred to the Schmahmann et al. (2000)

A highly similar pattern of stronger functional coupling within the cerebellum [84 voxels, *p* = 0.06, right dentate nucleus (8, − 60, − 30)] in the CH group than in the AD group was also observed when we used another cerebellar seed region of lobule VI [coordinates (20, − 48, − 22); right-middle panel in Fig. 3b but not presented in Table 4]. With respect to this seed region, we also found that the right fastigial nucleus (2, − 56, − 26) was more strongly coupled within the cerebellum in the ADO group than in the AD group (Table 4 and the rightmost panel in Fig. 3b).

In favor of these results, when we examined the functional connectivity from the ipsilateral paravermal region [lobule V; coordinates (8, − 56, − 10)], we found a cluster of active voxels (50 voxels) that exhibited greater within-cerebellar coupling [lobule VI (− 18, − 48, − 14)] in the CH group than in the AD group (violet section in the right-middle panel in Fig. 3c).

Hence, this series of results clearly indicated that functional coupling within the cerebellum was stronger in the younger (CH and ADO) groups than in the AD group. It appears that long-range cerebrocerebellar connectivity is stronger in adults, whereas within-cerebellar local connectivity is stronger in children and even in adolescents.

### “Local-to-distant” development of the cerebrocerebellar sensorimotor network

When we plotted individuals’ within-cerebellar connectivity against their cerebrocerebellar distant connectivity (Fig. 4), we could clearly observe the “local-to-distant” developmental dynamics of the human cerebrocerebellar sensorimotor network. In general, children exhibited relatively higher within-cerebellar and lower long-range cerebrocerebellar connectivity, whereas adults exhibited relatively higher long-range cerebrocerebellar connectivity with relatively lower but stable within-cerebellar connectivity. The adolescent data were generally located between the child and adult data, although there were some individual differences.

Hence, it appeared that the human cerebrocerebellar sensorimotor network develops by reducing within-cerebellar local connectivity and increasing long-range cerebrocerebellar connectivity, and that this functional development proceeds gradually from childhood to adulthood, which fits well with the hypothesis that the typically developing human brain develops by decreasing short-range connections and increasing long-range connections (Fair et al. 2007).

### Diffusion MRI

**Fig. 5:**
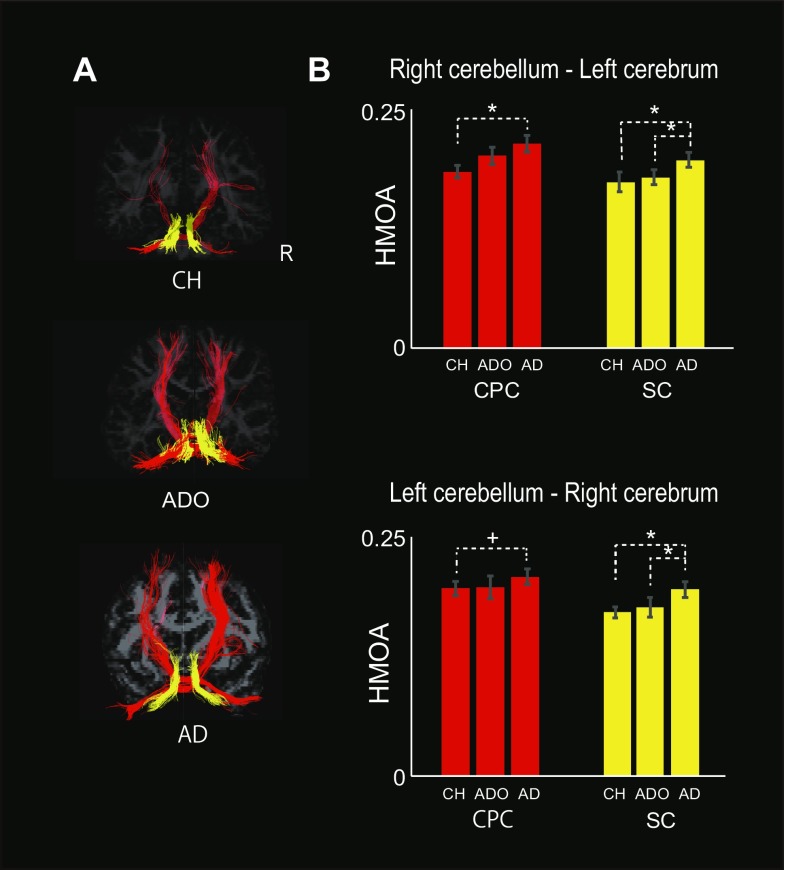
The tractography of each tract in a representative participant (**a**) and the mean hindrance-modulated orientational anisotropy (HMOA) value across participants in each group for the cortico-ponto-cerebellar (CPC) and superior cerebellar (SC) tracts (**b**). **a** The tractography of each tract (right-cerebellum–left-cerebrum and left-cerebellum–right-cerebrum of the CPC or SC tracts) in a representative participant from each group (CH, ADO, and AD from the top to bottom panels). The hemisphere on the right is the right hemisphere. Red tracts indicate the CPC tracts and yellow tracts indicate the SC tracts that connect the right-cerebellum and left-cerebrum and the left-cerebellum and right-cerebrum. **b** The mean HMOA value across participants in each group (vertical axis) is indicated for each tract (red bars for CPC and yellow bars for SC). The upper panel presents the data obtained from the tractography that connects the right cerebellum and the left cerebral cortex (cerebrum), and the lower panel presents the data obtained from the tractography that connects the left cerebellum and the right cerebrum. A small line mounted on each bar indicates the standard error of the mean across the participants in each group. A significant group difference is indicated by an asterisk above the graph (family-wise error-corrected for multiple comparisons, *p < 0.05, ^+^*p* < 0.1). *AD* adult, *ADO* adolescent, *CH* child

In the anatomical study, we examined the degree of fiber maturity in the cerebrocerebellar afferent and efferent tracts from childhood to adulthood. We could reconstruct the tractography of each tract in all participants. The tractography of each tract obtained in a representative participant in each group is presented in Fig. 5a.

In the CPC tract, the one-way ANOVA revealed a significant difference across the groups in the tract connecting the right cerebellum and the left cerebral cortex [*F*(2, 56) = 4.135, *p* = 0.021: top panel in Fig. 5b]. The post hoc analysis revealed significantly lower HMOA values in the CH group than in the AD group (*p* = 0.018). The same ANOVA also revealed a significant trend of group differences in the tract connecting the left cerebellum and the right cerebrum [*F*(2, 56) = 2.809, *p* = 0.069: bottom panel in Fig. 5b] The post hoc analysis revealed a significant trend of lower HMOA values in the CH group than in the AD group (*p* = 0.069).

In the SC tract, significant group differences were found in the tracts of both hemispheres, which connect the right (left) cerebellum and the left (right) cerebrum [the right-to-left SC: *F*(2, 56) = 7.697, *p* = 0.001; the left-to-right SC: *F*(2, 56) = 7.081, *p* = 0.002; Fig. 5b]. The post hoc analysis revealed significantly lower HMOA values for the SCs of both hemispheres not only in the CH group, but also in the ADO group than in the AD group (*p* = 0.002 between CH and AD; *p* = 0.013 between ADO and AD for the right-to-left SC, *p* = 0.002 between CH and AD; *p* = 0.024 between ADO and AD for the left-to-right SC).

While significant differences in HMOA values were observed in CH vs. AD and in ADO vs. AD, no significant differences were found between CH and ADO groups in any tracts.

The results indicate that cerebrocerebellar anatomical connections are still under development during childhood and seem to gradually mature with development (Fig. 5b).

Finally, we found no significant correlations between the effect-size of long-range cerebrocerebellar functional connectivity (in any of cerebellar seed regions) and the HMOA value in either the right-cerebellum-to-left-cerebrum CPC or the SC tract, even when we examined the correlations at the entire population level (*n* = 57) or in each group separately.

## Discussion

In the present study, we demonstrated both functional development of the cerebrocerebellar sensorimotor network and anatomical (structural) development of the cerebrocerebellar circuit in the typically developing human brain. During a right-hand motor task, we found stronger functional connectivity in the sensorimotor domain of the long-range cerebrocerebellar network in adults, whereas more locally-augmented, within-cerebellar connectivity was observed in children and adolescents. The weaker long-range cerebrocerebellar connectivity in children and adolescents seemed to have some relationship with the lesser degree of fiber maturity in the cerebrocerebellar afferent and efferent tracts. Hence, adult-like cerebrocerebellar functional coupling is not yet achieved during childhood and adolescence even for fundamental sensorimotor brain functions probably due to the immaturity of anatomical maturation of the cerebrocerebellar tracts. This study clearly demonstrated the principle of “local-to-distant” development of the functional brain network in the human cerebrocerebellar sensorimotor network, which might be associated with the slow anatomical maturation process in the long-range cerebrocerebellar pathway.

### fMRI study

The present right-hand motor task consistently activated the sensorimotor brain structures across all age groups (Fig. 2a). This indicated the fundamental importance of these structures when performing a motor task, regardless of developmental stages.

On the other hand, adults exhibited greater activity in the cerebrocerebellar sensorimotor network (Fig. 2b). The greater activity in the contralateral SM1 seems to be incompatible with previous reports of greater contralateral SM1 activation in children than in adults during finger-tapping tasks using their right fingers (De Guio et al. 2012; Turesky et al. 2018). We may attribute this discrepancy to differences in the task. Both of the present and previous tasks included a timing motor-control component because the tasks consistently asked the participants to generate a movement at a given time [1 Hz in the present study, 2 Hz in De Guio et al. (2012), and either 0.87, 1.11, or 1.54 Hz in the study by Turesky et al. (2018)]. On the other hand, our task required the participants to keep controlling the alternating extension–flexion movements of the wrist, while the previous tasks required simple button presses with the index finger (De Guio et al. 2012) or the thumb (Turesky et al. 2018). Thus, the range of motion appeared to be larger in our task, and the participants had to continuously control wrist extension and flexion movements alternatingly by predicting and adjusting movement time within the range of motion (between 0° and 60°) to touch either stopper in precise synchronization with 1-Hz audio tones (Fig. 1). Thus, the main difference between the tasks is that the present task required predictive sensory-motor control and online feedback control of the wrist, while the previous tasks required more simple generation of finger movement as a response. Hence, the present task likely required more sensory-motor association components.

This view seems to be in line with the fact that the presently observed greater activity in adults was located mainly in the postcentral gyrus and in the cortices lining the postcentral sulcus, which can be considered higher-order somatosensory and sensorimotor-association areas (Iwamura et al. 1994; Naito et al. 2005, 2008) (Fig. 2b). Furthermore, this view was further supported by additional evidence. Namely, when we examined functional connectivity with the left M1 [area 4a; coordinates (− 30, − 26, 62)], we found a cluster of active voxels (nine voxels) in the left SI [area 1 (− 36, − 36, 68)], which exhibited stronger functional coupling with M1 in the AD group than in the CH group (not provided in the Fig). The location of this SI activity highly corresponded to the SI region (− 36, − 40, 68) that was active during the right-wrist movement in adults in our previous study (Amemiya and Naito 2016). Thus, it appeared that the adults’ M1 communicated more strongly with the SI than did the children’s during the motor task, suggesting that cortical sensory-motor association function is more well-developed in adults than in children.

We found stronger long-range cerebrocerebellar functional connectivity in adults than in children (Fig. 3). One may argue that the greater cerebrocerebellar sensorimotor activity in adults (Fig. 2b) might cause this result. However, we also found stronger long-range cerebrocerebellar connectivity in adults than in adolescents (left-middle panel in Fig. 3), who exhibited no significant group difference compared to adults. In addition, when compared to adults, we found stronger within-cerebellar connectivity in children and adolescents (Fig. 3b), who exhibited no significantly greater activity than adults. Thus, we may assume that the present between-group difference in connectivity likely reflects more augmented neuronal communication in one group than in other groups, which cannot be explained merely by between-group differences in the degree of brain activation.

Many previous studies have suggested that the human cerebrocerebellar sensorimotor network is involved in online feedback control, predictive (timing) control of movements, and coordinated muscle control etc. (Cohen et al. 2017; Ivry 2003; Ivry and Spencer 2004; Sokolov et al. 2017). Thus, it is likely that the adult brain, which has stronger connectivity in the long-range cerebrocerebellar sensorimotor network, is capable of performing more sophisticated motor control in terms of the above aspects e.g. less variable timing motor control; (Naito et al. 2016). The lack of behaviorally detailed data in the present study makes us difficult to draw this conclusion; however, we can address this issue in our future studies.

In contrast to the adult brain, we found increased local functional connectivity within the cerebellum in children and adolescents (Fig. 3; Table 4), particularly in the cerebellar nuclei. These nuclei can be considered the cerebellar output channels (Hoover and Strick 1999; Middleton and Strick 2001), particularly the dentate nucleus as the main cerebellar output channel towards the cerebrum via the thalamus (Hoover and Strick 1999; Middleton and Strick 2001; Strick et al. 2009). Thus, the evidence that these nuclei were more strongly coupled with the ipsilateral cerebellar hemisphere (lobule VI) in children and adolescents than in adults (Table 4; Fig. 3) directly indicates increased functional connectivity within the local cerebellar circuit in the younger groups.

### Diffusion MRI study

Non-human primate studies have demonstrated that the cerebellum anatomically connects to the various cerebral regions that are the source of input to the cerebellum in a topographically organized manner by forming cerebrocerebellar closed loops (Strick et al. 2009). Such closed loops are formed in parallel between particular sections of the cerebellum and the motor (Dum and Strick 2003; Middleton and Strick 2000; Strick et al. 2009), prefrontal (Kelly and Strick 2003; Middleton and Strick 2000; Strick et al. 2009), and parietal areas (Clower et al. 2005; Dum and Strick 2003), and so on.

However, in the present study, we could not consider such topographical organization when we reconstructed the CPC afferent tract and the SC efferent tract. Rather, we simply summed the fiber streamlines passing through the two ROIs for each tract. Thus, in the present study, we depicted more general cerebrocerebellar afferent and efferent tracts, but could not depict these tracts selectively connecting between the cerebellum and the sensorimotor areas (cerebrocerebellar tracts in sensorimotor domain). Hence, the present lower HMOA value in children could generally reflect less structural maturity of the cerebrocerebellar tracts, which target not only the sensorimotor regions, but also the prefrontal, parietal and other regions. If we consider the fact that the human cerebellum contributes not only to sensorimotor functions, but also to a variety of cognitive (non-motor) functions, such as language, working memory, attention set shifting etc. (De Smet et al. 2013; Schmahmann and Sherman 1998; Stoodley and Schmahmann 2010; Rapoport et al. 2000) via cerebrocerebellar interactions (Buckner et al. 2011; Kipping et al. 2013; Toro et al. 2008), the less the structural maturity of cerebrocerebellar tracts in children might be associated with their immaturity not only in sensorimotor functions (Barnett et al. 2010; Davies and Rose 2000; Dayanidhi et al. 2013; Janacsek et al. 2012; Krombholz 1997), but also in various cognitive functions (Blakemore and Choudhury 2006; Bolduc et al. 2012; Diamond 2000; Tavano et al. 2007).

It has been demonstrated that cerebellar white matter develops rapidly and drastically within the first 3 years of life (Pieterman et al. 2017; Saksena et al. 2008), and that its development becomes relatively stable from childhood to adolescence (9–17 years) (Leitner et al. 2015). However, in the latter study, cerebellar maturation was investigated up to the period of adolescence but not in the adulthood, and thus, it was unclear whether the maturity of the cerebellar tracts in children is comparable to that of adult tracts. In addition, this study (Leitner et al. 2015; Saksena et al. 2008) employed DTI, and used FA as an anisotropy index. While DTI is a useful method, the tensor model has technical limitations. This model assumes one major fiber direction even when a voxel contains fibers of multiple orientations. Thus, the FA value is disadvantageous for precisely describing the diffusivity of water molecules within fibers of complex orientation.

In the present study, we used an SD approach and evaluated a novel anisotropy index of HMOA, and demonstrated that the HMOA values of the cerebrocerebellar afferent and efferent tracts are significantly lower in children than in adults, and gradually increased with development (Fig. 5b). Thus, this is the first study to demonstrate the structural development of human cerebrocerebellar afferent and efferent tracts from childhood to adulthood in the typically developing human brain. Since a lower HMOA value is most likely associated with a lesser degree of myelination, lower axonal density, smaller axonal diameter, or wider fiber orientation dispersion and so on, our results indicate that the cerebrocerebellar afferent and efferent tracts are still under development during childhood (8–11 years) and gradually mature along with development.

It has been postulated that white matter, in general, undergoes continuous changes throughout life since its growth is activity-dependent (Fields 2015). The cerebellum is located at a distance from the cerebral cortex, therefore, maturation of cerebrocerebellar anatomical (structural) connections most likely takes a considerable amount of time. Similarly, when we examined the degree of fiber maturity of the spinocerebellar (SpC) tract in the present participants (see Supplementary Information), which is involved in the processing of somatosensory (proprioceptive and cutaneous) afferent inputs from the body via the spinal cord to the cerebellum (Bosco and Poppele 2003; Grant 1982; Cohen et al. 2017), even in the SpC tract that mediates fundamental sensory processing, we found significantly lower HMOA values in children than in adults (Supplementary Fig. 1). It is known that the vermis and intermediate sections of the cerebellum (spinocerebellum) receive the somatosensory afferent inputs, and the spinocerebellum is considered to be a phylogenetically (and ontogenetically) older region in the cerebellum (Tiemeier et al. 2010). Thus, our finding suggests that even this “older” SpC tract involving fundamental sensorimotor functions develops slowly, likely owing to the long distance between the spinal cord and the cerebellum and to accumulating physical activity experience from childhood to adulthood. This view seems to be compatible with previous evidence that the corticospinal (long motor-descending) tract also continuously develops and that the maximal level of FA for this tract is sufficiently achieved after 20 years of age (Lebel et al. 2012; Peters et al. 2014).

## Conclusion

In the present study, we demonstrated that, in the typically developing human brain, functional connectivity is stronger in the long-range cerebrocerebellar sensorimotor network in adults, whereas it is more locally-augmented within the cerebellum in children and adolescents when they perform a right-hand motor task requiring predictive sensory-motor and online feedback control (Fig. 3). We also demonstrated that the cerebrocerebellar fiber tracts are still under development during childhood (8–11 years) and gradually mature with age (Fig. 5). The series of results suggested that adult-like use of the cerebrocerebellar distant network slowly matures even for fundamental sensorimotor functions and that the maturation process progresses by decreasing short-range and increasing long-range functional connections (Fig. 4; cf. Fair et al. 2007). Hence, our study clearly demonstrated the principle of “local-to-distant” development of functional brain networks in the human cerebrocerebellar sensorimotor network during a motor task (cf. Fair et al. 2009; Dosenbach et al. 2010), which might be associated with the slow anatomical maturation process of the long-distance cerebrocerebellar pathway. Finally, the present study also provides valuable baseline information for future investigations of cerebrocerebellar development in healthy children and in clinical pediatric populations, e.g. those with developmental coordination disorder (Gomez and Sirigu 2015; Ivry 2003; Zwicker et al. 2012).

## Electronic supplementary material

Below is the link to the electronic supplementary material.

Supplementary material 1 (EPS 3113 KB)

Supplementary material 2 (DOCX 20 KB)
