# Supplementary material for: Local-to-distant development of the cerebrocerebellar sensorimotor network in the typically developing human brain: a functional and diffusion MRI study
Source: Brain Struct Funct. 2019 Feb 7;224(3):1359–75. doi: 10.1007/s00429-018-01821-5 (PMC6499876; doi:10.1007/s00429-018-01821-5)
Supplement: Supplementary file 2 — Supplementary material 2 (DOCX 20 KB) [file 429_2018_1821_MOESM2_ESM.docx]

Title: Local-to-distant development of the cerebrocerebellar sensorimotor network in the typically developing human brain: a functional and diffusion MRI study

Journal: *Brain Structure & Function*

Authors: Kaoru Amemiya^1^, Tomoyo Morita^1,2^, Daisuke N. Saito^3,4,5^, Midori Ban^6^, Koji Shimada^3,4^, Yuko Okamoto^3,7^, Hirotaka Kosaka^3,4,8^, Hidehiko Okazawa^3,4^, Minoru Asada^1,2^, and Eiichi Naito^1,9^

Affiliations:

^1^Center for Information and Neural Networks (CiNet), National Institute of Information and Communications Technology (NICT), 1-4 Yamadaoka, Suita, Osaka 565-0871, Japan

^2^Graduate School of Engineering, Osaka University, 2-1 Yamadaoka, Suita, Osaka 565-0871, Japan

^3^Research Center for Child Mental Development, University of Fukui, 23-3 Matsuoka-shimoaizuki, Eiheiji, Yoshida, Fukui 910-1193, Japan

^4^Biomedical Imaging Research Center, University of Fukui, 23-3 Matsuoka-shimoaizuki, Eiheiji, Yoshida, Fukui 910-1193, Japan

^5^Research Center for Child Mental Development, Kanazawa University, 13-1 Takaramachi, Kanazawa, Ishikawa 920-8640, Japan

^6^Graduate School of Engineering Science, Osaka University, 1-3, Machikaneyama, Toyonaka, Osaka 560-8531, Japan

^7^ATR promotions, 2-2, Hikaridai, Seika, Soraku-gun, Kyoto 619-0288, Japan ^8^Department of Neuropsychiatry, Faculty of Medical Sciences, University of Fukui, 23-3 Matsuoka-shimoaizuki, Eiheiji, Yoshida, Fukui 910-1193, Japan,

^9^Graduate School of Frontier Biosciences, Osaka University, 1-3 Yamadaoka, Suita, Osaka 565-0871, Japan

Correspondence: Eiichi Naito, PhD

Email: [eiichi.naito@nict.go.jp](mailto:eiichi.naito@nict.go.jp)

Supplementary information

In the present diffusion MRI study, we also evaluated the degree of fiber maturity of the spinocerebellar tract (SpC) in the present participants. It is known that the vermis and intermediate sections of the cerebellum receive various somatosensory (proprioceptive and cutaneous) afferent inputs from the body via the spinal cord in primates. This pathway is called the spinocerebellar tract (SpC), and it plays an important role in the processing of somatosensory information to control body parts and body posture (Bosco and Poppele 2001; 2003; Cohen et al. 2017; Grant 1982). In addition to the CPC and SC tracts, we examined the extent of fiber maturity of the SpC using the same approach described in the text. We set the ROIs for the SpC tract around the cerebellar nuclei and the central part of the inferior cerebellar peduncle. This tract most likely includes the afferent fibers from the spinal cord and the olivary nucleus into the cerebellum.

We were able to reconstruct the tractography of each SpC tract in all participants (Supplementary Figure 1A). The one-way ANOVA showed significant group differences in HMOA value in both the left and the right tracts (left SpC: *F*(2, 56) = 7.106, *p* = 0.002, right SpC: *F*(2, 56) = 3.411, *p* = 0.04; Supplementary Figure 1B). The post-hoc analysis revealed significantly lower HMOA values for bilateral tracts in the CH group compared with those of the AD group (*p* = 0.009 for the left SpC, *p* = 0.0037 for the right SpC). A significantly lower value was also observed for the left SpC in the ADO group compared with the AD group (*p* = 0.004).

In the present study, we only targeted the dorsal and rostral SpC pathways when setting the two ROIs (see methods and Catani et al. 2008), though an animal (cat) study has suggested that the SpC tract consists of at least three (dorsal, ventral, and rostral) pathways (Grant 1982). We did not target the ventral SpC pathway because the reconstruction of the ventral pathway is technically complicated, given that it passes through the superior cerebellar peduncle at a very narrow site. Despite these technical limitations, we could depict the general features of fiber streamlines in the SpC tracts.

The spinocerebellum is considered to be a phylogenetically (and ontogenetically) older region of the cerebellum (Tiemeier et al. 2010). Thus, our findings suggest that even such ‘older’ spinocerebellar tracts involving ‘fundamental’ sensorimotor function develop slowly, probably owing to the long distance between the spinal cord and the cerebellum, and the accumulation of physical activity experience from childhood to adulthood.

Figure legends

Supplementary Figure 1

Tractography of the right and left spinocerebellar (SpC) tracts of a representative participant (A) and mean HMOA value of participants in different age groups (B). A: Tractography of the right and left SpC tracts (green) in a representative participant from each group (CH, ADO, and AD from top to bottom panel). The right hemisphere shown on the right. B: Mean HMOA values of participants in each group for the right (upper) and left (lower) SpC tracts. Error bars indicate the standard error of the mean across the participants in each group. Significant differences between groups are indicated by asterisks (family-wise error corrected for multiple comparisons, * *p* < 0.05). AD: adult, ADO: adolescent, CH: child.

*Reference*

Bosco G, Poppele RE (2003) Modulation of dorsal spinocerebellar responses to limb movement. II. Effect of sensory input. J Neurophysiol 90:3372-3383. doi:10.1152/jn.00204.2003

Bosco G, Poppele R (2001) Proprioception from a spinocerebellar perspective. Physiol rev 81 (2):539-568. doi:10.1152/physrev.2001.81.2.539

Catani M, Jones DK, Daly E, Embiricos N, Deeley Q, Pugliese L, Curran S, Robertson D, Murphy DG (2008) Altered cerebellar feedback projections in Asperger syndrome. Neuroimage 41:1184-1191. doi:10.1016/j.neuroimage.2008.03.041

Grant G (1982) Spinocerebellar connections in the cat with particular emphasis on their cellular origin. Exp Brain Res Suppl. 6:466-476.

Tiemeier H, Lenroot RK, Greenstein DK, Tran L, Pierson R, Giedd JN (2010) Cerebellum development during childhood and adolescence: a longitudinal morphometric MRI study. Neuroimage 49:63-70. doi:10.1016/j.neuroimage.2009.08.016
